# Identification of hub cuproptosis related genes and immune cell infiltration characteristics in periodontitis

**DOI:** 10.3389/fimmu.2023.1164667

**Published:** 2023-05-05

**Authors:** Shuying Liu, Jiaying Ge, Yiting Chu, Shuangyu Cai, Aixiu Gong, Jun Wu, Jinghan Zhang

**Affiliations:** ^1^ Department of Stomatology, Children’s Hospital of Nanjing Medical University, Nanjing, Jiangsu, China; ^2^ Department of Anatomy, Histology and Embryology, Nanjing Medical University, Nanjing, Jiangsu, China; ^3^ Department of Obstetrics and Gynecology, Nanjing Drum Tower Hospital, Nanjing University Medical School, Nanjing, Jiangsu, China

**Keywords:** cuproptosis, hub, immune, infiltration, periodontitis

## Abstract

**Introduction:**

Periodontitis is an inflammatory disease and its molecular mechanisms is not clear. A recently discovered cell death pathway called cuproptosis, may related to the disease.

**Methods:**

The datasets GSE10334 of human periodontitis and control were retrieved from the Gene Expression Omnibus database (GEO) for analysis.Following the use of two machine learning algorithms, least absolute shrinkage and selection operator (LASSO) and support vector machine-recursive feature removal (SVM-RFE) were used to find CRG-based signature. Then the Receiver operating characteristic (ROC) curves was used to evaluate the gene signature's discriminatory ability. The CIBERSORT deconvolution algorithm was used to study the link between hub genes and distinct types of immune cells. Next, the association of the CRGs with immune cells in periodontitis and relevant clusters of cuproptosis were found. The link between various clusters was ascertained by the GSVA and CIBERSORT deconvolution algorithm. Finally, An external dataset (GSE16134) was used to confirm the diagnosis capacity of the identified biomarkers. In addition, clinical samples were examined using qRT-PCR and immunohistochemistry to verifiy the expression of genes related to cuprotosis in periodontitis and the signature may better predict the periodontitis.

**Results:**

15 periodontitis-related DE-CRGs were found,then 11-CRG-based signature was found by using of LASSO and SVM-RFE. ROC curves also supported the value of signature. CIBERSORT results of immune cell signature in periodontitis showed that signature genes is a crucial component of the immune response.The relevant clusters of cuproptosis found that the NFE2L2, SLC31A1, FDX1,LIAS, DLD, DLAT, and DBT showed a highest expression levels in Cluster2 ,while the NLRP3, MTF1, and DLST displayed the lowest level in Cluster 2 but the highest level in Cluster1. The GSVA results also showed that the 11 cuproptosis diagnostic gene may regulate the periodontitis by affecting immune cells. The external dataset (GSE16134) confirm the diagnosis capacity of the identified biomarkers, and clinical samples examined by qRT-PCR and immunohistochemistry also verified that these cuprotosis related signiture genes in periodontitis may better predict the periodontitis.

**Conclusion:**

These findings have important implications for the cuproptosis and periodontitis, and highlight further research is needed to better understand the mechanisms underlying this relationship between the cuproptosis and periodontitis.

## Introduction

Periodontitis is an extremely common chronic inflammatory illness that affects the tooth’s supporting structures (1, 2). By interfering with the balance of pro- and anti-inflammatory responses in periodontium, the aberrant inflammation process causes processes like gingival recession and alveolar bone loss, which result in the death of tooth-supporting tissues (3). Many people around the world suffer from periodontitis. However, the molecular mechanisms by which pathogens and risk factors damage the periodontium remain unknown.

Based on the mechanism of occurrence, different cell death patterns existed, such as apoptosis, ferroptosis, pyroptosis, and necroptosis. Previous research has shown that cells in the periodontal ecological niche may respond with an inflammatory response or cell death in reaction to significant changes in the extracellular environment, alerting the immune defense system (3). The diseased periodontal tissues show decreased apoptosis (4), while pyroptosis and necroptosis are increased in inflamed periodontal tissues (5, 6). Periodontal ligament fibroblasts may undergo ferroptosis as a result of activation of NCOA4-mediated ferritinophagy, which can compromise iron homeostasis (7).

Recently, cuproptosis (8), a brand-new cell death process, has come to our attention. Cuproptosis demonstrated that the interaction of lipoylated tricarboxylic acid (TCA) cycle components with excess copper can cause proteotoxic stress and cell death (8). Stimulated inflammatory conditions raise serum copper levels, cause oxidative stress, and activate the inflammatory response (9). The cytotoxicity caused by copper may also be accelerated by inflammation (10). However, the regulating genetic and molecular mechanisms underlying cuproptosis in periodontitis etiology and systemic complications are largely unknown.

As a result, the current study aimed to use publicly available transcriptomics datasets for integrative bioinformatics to identify a cuproptosis-related gene signature and its functional pathways in periodontitis, thereby increasing our understanding of potential cuproptosis-related mechanisms in periodontal diseases. Our study may reveal crucial pathogenic pathways and therapeutic targets for periodontitis, and they may also provide guidelines for further experimental research.

## Materials and methods

### Data processing

Datasets about periodontitis, including GSE10334 and GSE16134, were collected from the GEO database. By accessing earlier literature, the gene set related to cuproptosis was discovered (11).

### Screening of cuproptosis-associated genes in periodontitis patients

Differentially expressed (DE)-CRGs were identified using the R package “Linear Models for Microarray Data” (“limma”) (12), with a P-value<0.05 and |log FC| > 0 as the cutoff. A heat map was used to show them. The GSE10334 dataset was used to identify the CRGs’ expression patterns in patients and healthy controls, which were then further confirmed using the GSE16134 dataset. Using Cytoscape 3.9.1 and String database (https://string-db.org), we created a PPI (protein-protein interaction) network to evaluate gene interactions among the 15 DE-CRGs. To evaluate the correlation between CRG pairs, the Pearson’s correlation coefficient (13) was computed for the DE-CRGs in periodontitis samples from GSE10334 and visualized using “corrplot” in R.

### A Diagnostic model based on CRGs related to periodontitis was constructed and validated

The least absolute shrinkage and selection operator (LASSO) model and support vector machine recursive feature elimination (SVM-RFE) (14, 15) were used to pick the most relevant DE-CRGs (16), and minimum lambda is defined as the ideal value. On the basis of the chosen diagnosistic signs, multivariate logistic regression analysis was then used to create a diagnostic model. By using the “pRPC” package in R, the prediction accuracy of the training set and the test set was assessed by plotting a receiver operating characteristic (ROC) curve. A diagnosis model was subsequently developed using multivariate logistic regression analysis based on the predetermined diagnosistic indications. The prediction accuracy of the training set and the test set was evaluated by producing a receiver operating characteristic (ROC) curve (17) using the “pRPC” package (18) in R.

### Analysis of immune cell infiltration by CIBERSORT

Using CIBERSORT(https://cibersortx.stanford.edu) algorithm, the differential immune cell infiltration was evaluated (19). This approach made use of the default signature matrix, which contains 1000 permutations. Only data with deconvolution p < 0.05 were kept in order to boost the reliability of the findings. To determine the differences between the various clusters and the immune cell infiltration between the periodontitis and normal groups, the Wilcoxon test was used.

The correlations between immune cell subtypes and the predicted amounts of key genes were calculated using the “corrplot” R package (Spearman’s rank correlation method). For displaying the results, the R packages “ggplot2” and “ggpubr” were utilized.

### Consensus clustering analysis

Different cuproptosis-related molecular patterns were identified by using consensus clustering. The consensus clustering approach, which makes use of the “ConsensuClusterPlus” package, was used to calculate the quantity of unsupervised clusters and their stability (20). Strong intraclass links and weak interclass relationships, as well as the absence of any groups with unacceptable small sample sizes, were the criteria for clustering. The cumulative distribution function (CDF) curve first increased gradually. The subtype assignments were checked using PCA. The gene expression profiles of the diagnosistic DE-CRGs within various clusters were compared using the R package “ggpubr”, and DE-PRGs with a P-value <0.001 were taken into consideration as the specific diagnostic CRGs.

### Annotation of gene enrichment functions and gene set variation analysis

GSVA (21), a nonparametric, unsupervised approach for calculating gene set enrichment through expression profiles, was used to identify differences between various biological process clusters.

### Establishment and validation of nomogram

Discriminative power of nomogram is evaluated *via* the area under the ROC(receiver operating characteristic curve), which is created using the rm packages. Both internally and externally, in the development cohort and validation cohort, the nomogram was validated. The degree of agreement between expected probability and actual results were evaluated by calibration curves (22). To evaluate the nomogram’s clinical usefulness and advantages, decision curve analysis (DCA) was used (23).

### Tissue collection and ethics statement

20 patients (10 males, 10 females; Average age:40 ± 15 years) were included in this study. In the periodontitis group, gingival margin epithelial tissue and periodontal pocket connective tissue were included, were collected during clinical crown lengthening, curettage, and tooth extraction. In the control group, gingival tissues also including gingival margin epithelial tissue and periodontal connective tissue, were collected during clinical crown lengthening and tooth extraction. All patients met the following criteria: no antibiotics or anti-inflammatory drugs in the last 3 months, no periodontal treatment, and general health. Exclusion criteria include any systemic disease that may affect periodontal disease, immune deficiency, prior smoking history, or current smoking. All patients were informed and consented. Our study was approved by the Ethics Committee of Children’s Hospital of Nanjing Medical University (Nanjing, Jiangsu, PR China), and it was performed in compliance with the Declaration of Helsinki Principles.

### Real-time quantitative polymerase chain reaction

According to the manufacturer’s instruction, total RNA was extracted using TRIzol Reagent (Invitrogen, CA, USA) from samples of the periodontitis patient and normal periodontium. It was then purified using 75% ethanol, isopropanol and RNase-free water. Using HiScript® III All-in-one RT SuperMix Perfect, cDNA was created following the measurement of the RNA concentration and purity (Vazyme Biotech Co., Ltd.). The QuantStudio 3 Real-Time PCR System from Thermo Fisher Scientific was used to perform RT-qPCR with the ChamQ Universal SYBR qPCR Master Mix. The 2-DDCT mode was used to quantify the expression level. The reference gene for quantitative analysis was GAPDH. In **Supplementary Table 1**, the primer sequences for RT-qPCR are provided.

### Immunohistochemistry

After deparaffinization and dehydration, tissue sections were incubated overnight at 4°C with NLRP3(Novus Biologicals, NBP2-12446), DLST(Abcam, ab177934), SLC31A1 antibodies (Proteintech, 67221-1-Ig) after epitope retrieval, H_2_O_2_ treatment, and non-specific antigens blocking. Then the sections were treated for two hours at room temperature with secondary antibodies (Proteintech, SA00001-2), and an enhanced DAB staining kit (Vector, SK-4100) was used to detect the signal. The ImageJ software was used for histomorphometric evaluation.

### Statistical analysis

The graphs, calculations, and statistical analyses in qRT-PCR and IHC results were performed using GraphPad Prism software version 8.0 (San Diego, CA, USA).The means between the groups were compared using Student’s t test. Differences were considered significant at P < 0.05.

## Results

### Identifification of cuproptosis in periodontitis

**Figure 1:**
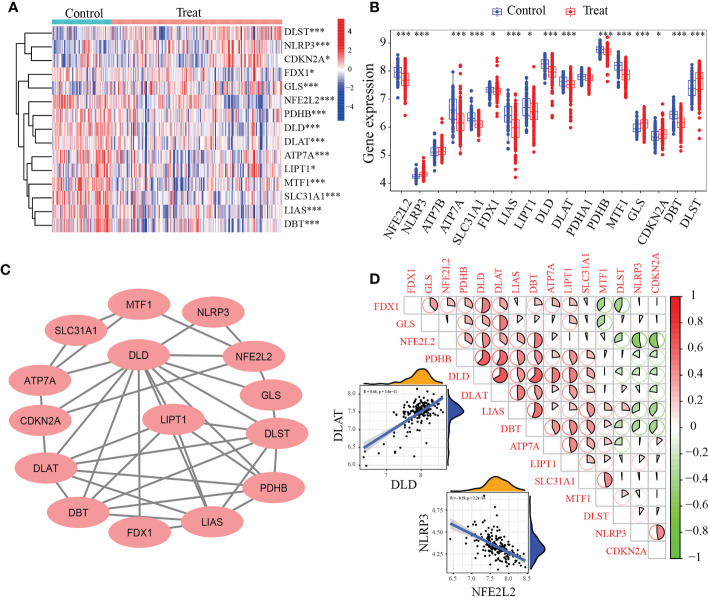
Identification of differentially expressed cuproptosis-related genes (DE-CRGs) in periodontitis and controls. **(A)** Heat map of 15 DE-CRGs in the periodontitis; **(B)** The box plots showed 15 CRGs differernt were expressed between periodontitis and healthy samples, while PDHA1 and ATP7B showed no difference; **(C)** Protein–protein interaction (PPI) network of the 15 DE-CRGs; **(D)** Correlations of the 15 DE-CRGs in the periodontitis samples Red, positive correlation; Green, negative correlation. The color depth and the size reflect the strength of the relevance. The strongest positive and the strongest negative correlation were displayed in scatter plots. *P < 0.05, ***P < 0.001.

Based on the GSE10334 dataset, a total of 15 DE-CRGs were identified (**Figures 1A, B**). Of these, 11 cuproptosis-related genes (CRGs)were down-regulated in periodontitis, including NFE2L2 (Nuclear factor erythroid 2-related factor 2, also know as NRF2), LIPT1(Lipoyltransferase 1), SLC31A1(Solute Carrier Family 31 Member 1), FDX1(Ferredoxin 1), LIAS(Lipoic Acid Synthetase), ATP7A (ATPase Copper Transporting Alpha), PDHB(Pyruvate Dehydrogenase E1 Subunit Beta), MTF1(Metal Regulatory Transcription Factor 1), DLD(Dihydrolipoamide dehydrogenase), DLAT(Dihydrolipoamide S-Acetyltransferase) and DBT(Dibutyltin), while 4 CRGs were up-regulated in periodontitis, including NLRP3(NLR Family Pyrin Domain Containing 3), GLS(Glutaminase), CDKN2A(Cyclin Dependent Kinase Inhibitor 2A) and DLST(Dihydrolipoamide S-Succinyltransferase).

The interactions of 15 periodontitis-related CRGs (**Figure 1C**) were depicted using the PPI network. The relevance of CUG pairs in periodontitis were demonstrated by correlation analysis (**Figures 1C, D**). DLD and DLAT had the strongest positive correlation (R = 0.46, p< 5.6e-11), while NFE2L2 and NLRP3 had the strongest negative correlation (R = -0.59, p <2.2e-16). These findings suggested that periodontitis is related to cuproptosis.

### Screening and validation of the key feature genes

**Figure 2:**
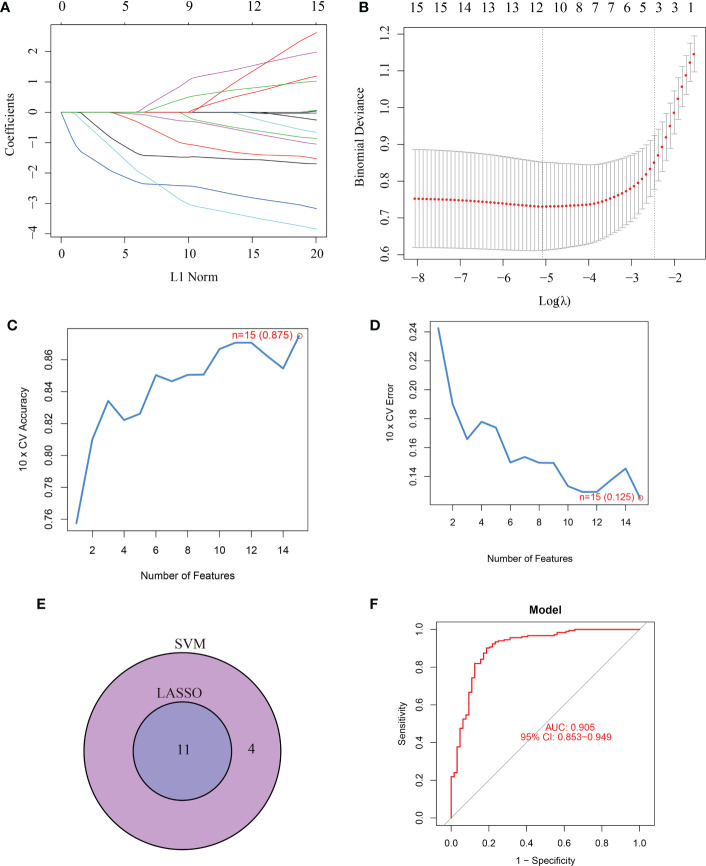
Screening for key feature genes by LASSO and SVM-RFE algorithms. **(A, B)** Feature genes identified using the least absolute shrinkage and selection operator (LASSO) logistic regression algorithm. Covariates are selected using the regularization parameter λ. **(C, D)** Support vector machine-recursive feature elimination (SVM-RFE) algorithm to screen feature genes. **(E)** Venn diagram demonstrating overlapping key feature genes screened by LASSO and SVM-RFE. **(F)** ROC curve.

15 CRGs were screened using LASSO analysis. Then, the optimum parameter was selected as 11 (**Figures 2A, B**). Similarly, the SVM-RFE algorithm discovered 15 feature genes from DE-CRGs (**Figures 2C, D**). 11 genes overlapped between the two algorithms, DLAT, NFE2L2, SLC31A1, NLRP3, LIAS, DLD, MTF1, DLST, GLS, FDX1 and DBT (**Figure 2E**), which were ultimately discovered as key feature periodontitis genes. Subsequently, the area under the ROC curve(AUC), was used to determine 11-CRGs as disease diagnostic genes, which showed that the AUC values of 11 CRGs were 0.905 (**Figure 2F**). The results indicated that the cuproptosis model should be taken into considering the diagnosis of periodontitis.

### Distinct immune characteristics characterized both periodontitis and controls

**Figure 3:**
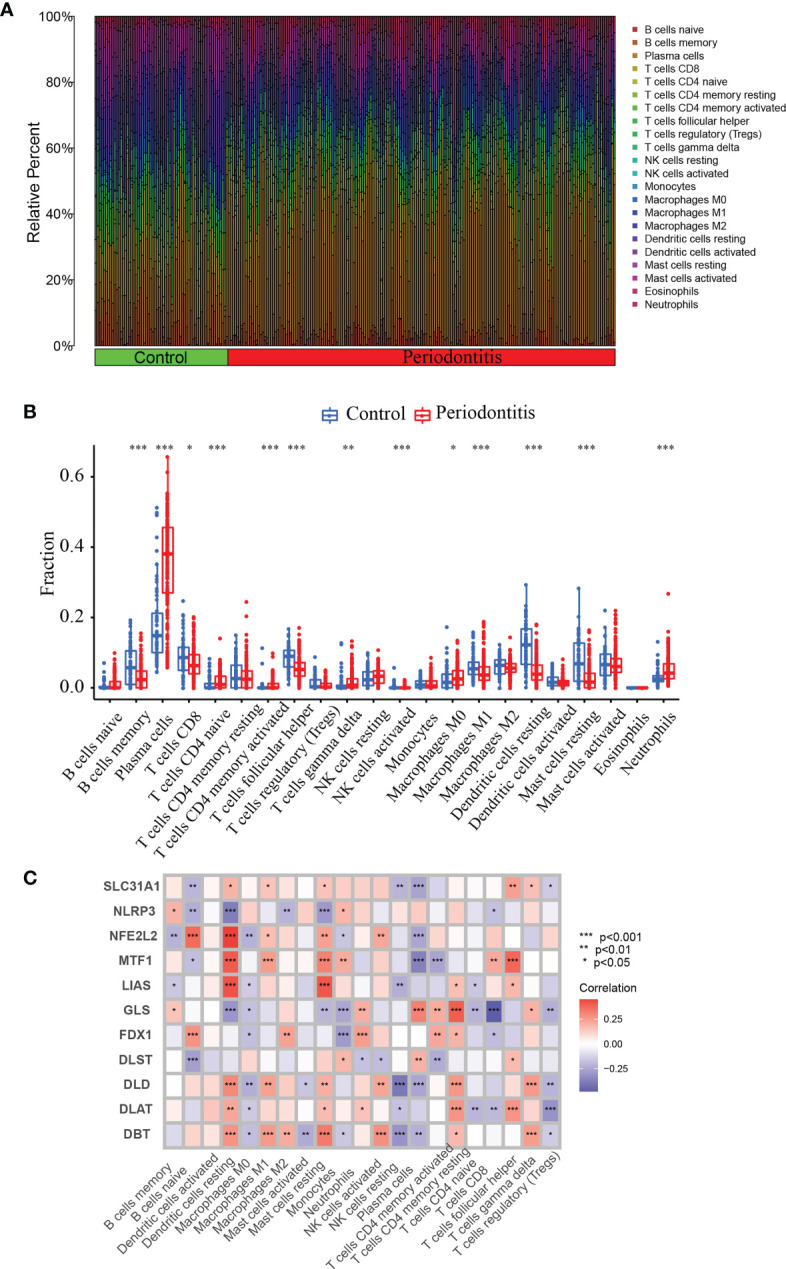
CIBERSORT immune infiltration analysis. **(A)** Percentage of immune cells. **(B)** Immune cell difference between periodontitis and normal control. **(C)** Correlation between 11 DE-CRGs and 22 immune cells. *P <0.05, **P < 0.01, ***P < 0.001.

Since periodontitis and immune response are strongly correlated. we used CIBERSORT to predict the pattern of immune cell signature in periodontitis. Percentages of the 22 types of immune cells in each sample were shown in **Figure 3A**.

To predict the pattern of immune cell signature in periodontitis, we used CIBERSORT to depict the percentages of the 22 different types of immune cells in each sample (**Figure 3A**). We also analyzed the immune characteristics differences between the periodontitis and control groups. There are significant difference in the levels of B memory cells, CD8^+^ T cells, plasma cells, navie CD4^+^ T cells, follicular helper T cells, memory activated CD4^+^ T cells, gamma delta T cells, activated NK cells, activated Eosinophils Mast cells, M1 Macrophages, M0 Macrophages, resting Dendritic cells, resting Mast cells and Neutrophils (**Figure 3B**). Furthermore, we performed correlation analysis of the 11 CRGs with immune cells in periodontitis. The correlation between the expression of these 11 genes and the immune cell ratio revealed that they were strongly associated with resting Dendritic cells, resting Mast cells, and plasma cells (**Figure 3C**). The findings revealed that the marker genes employed in the present diagnosis model is a crucial component of the immune response.

### Three clusters were discovered according to the 11 diagnostic DE-CRGs

**Figure 4:**
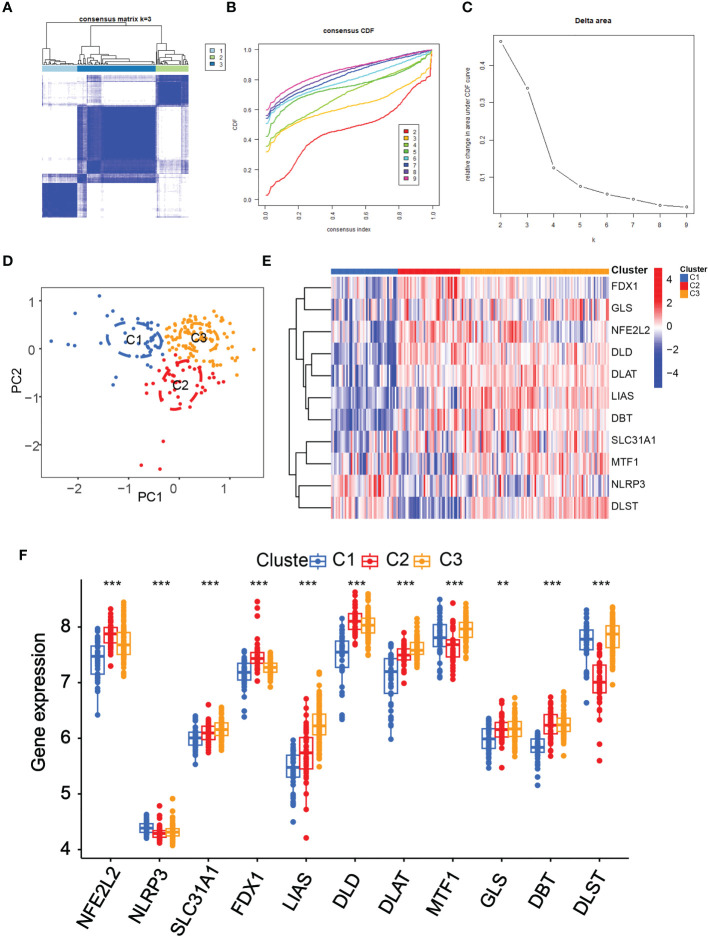
Identification of the cuproptosis-related clusters in periodontitis. Consensus clustering for the 183 periodontitis samples in GSE10334 based on the diagnosistic cuproptosis-related genes (PRGs). Three clusters were classified according to the consensus matrix **(A)**, consensus index of cumulative distribution function (CDF) **(B)**, and CDF delta area curve **(C)** for k = 3 by increasing the index from 2 to 9. **(D)** The principal component analysis (PCA) shows a different distribution of the three clusters. The expressions of the 11 diagnosistic CRGs in the three clusters are shown in the heat map **(E)** and box plots **(F)**. **P < 0.01, ***P < 0.001.

Subsequently, using an unsupervised consensus clustering technique, we classified samples with different cuproptosis patterns based on the expression of 11 cuproptosis regulators. The consensus matrix analysis revealed that k=3 was the ideal choice and that each sample in the cluster displayed a significant correlation (**Figures 4A–C**). The PCA revealed a distinction between the three clusters (**Figure 4D**). The heat map showed the 15 diagnostic CRGs gene expression profiles in the three clusters (**Figure 4E**).We also found that the NFE2L2, SLC31A1, FDX1, LIAS, DLD, DLAT and DBT showed a highest expression levels in Cluster2 than in Cluster1 (P-value<0.001)(**Figure 4F**). On the other hand, the NLRP3, MTF1, and DLST, displayed the lowest level in C2 but the highest level in C1 (P-value<0.001).

### Distinct biological functions enriched in the three clusters

**Figure 5:**
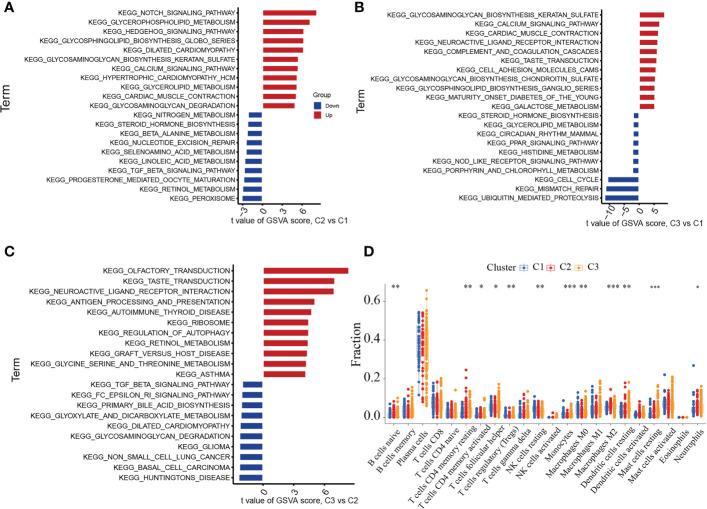
GSVA analysis among three cuproptosis clusters in the merged cohort. **(A)** GSVA analysis between cuproptosis cluster 1 and cluster 2. **(B)** GSVA analysis between cuproptosis cluster 1 and cluster 3. **(C)** GSVA analysis between cuproptosis cluster 2 and cluster 3. **(D)** CIBERSORT analysis between three distinct cuproptosis clusters based on immune-related gene expression. The asterisk symbol indicates the statistical p-value *p < 0.05; **p < 0.01; ***p < 0.001.

To identify the various biological processes connected to the three unique cuproptosis clusters in periodontitis, we used GSVA enrichment analysis. **Figure 5A** showed that cluster2 was highly enriched in several metabolic biological processes when compared to cuproptosis cluster1, including glycerophospholipid metabolism, nitrogen metabolism, selenoamino acid metabolism, linoleic acid metabolism, retinol metabolism and beta alanine metabolism. **Figure 5B** illustrated that comparing to cuproptosis cluster1, cuproptosis cluster 3 was markedly enriched in glycosaminoglycan biosynthesis keratan sulfate, neuroactive ligand receptor interaction, calcium signaling pathway, cardiac muscle contraction, complement and coagulation cascades and glycosaminoglycan biosynthesis chondroitin sulfate. Compared to cuproptosis cluster 2, **Figure 5C** indicated that cuproptosis cluster 3 was also significantly enriched in various immune activation pathways, including neuroactive ligand receptor interaction, autoimmune thyroid disease, graft versus host disease and asthma. Both cuproptosis cluster 1 and cuproptosis cluster 2 in periodontitis presented high metabolic characteristics. To further explore the relationship between immune cells and different m6A modification clusters, CIBERSORT was used to indicate the differences in almost all immune cells among the three cuproptosis clusters in the study. The cuproptosis cluster 2 tended to obtain the highest immune scores of activated mavie B cells, resting CD4 memory T cells, activated CD4 memory T cells, follicular helper T cells, regulatory T cells, gamma delta T cells, Monocytes, M0 Macrophages, M2 Macrophages, resting Dendritic cells, resting Mast cells and Neutrophils (**Figure 5D**). These results showed the 11 cuproptosis diagnostic gene may regulate the periodontitis by affecting immune cells.

### Nomogram construction and validation

**Figure 6:**
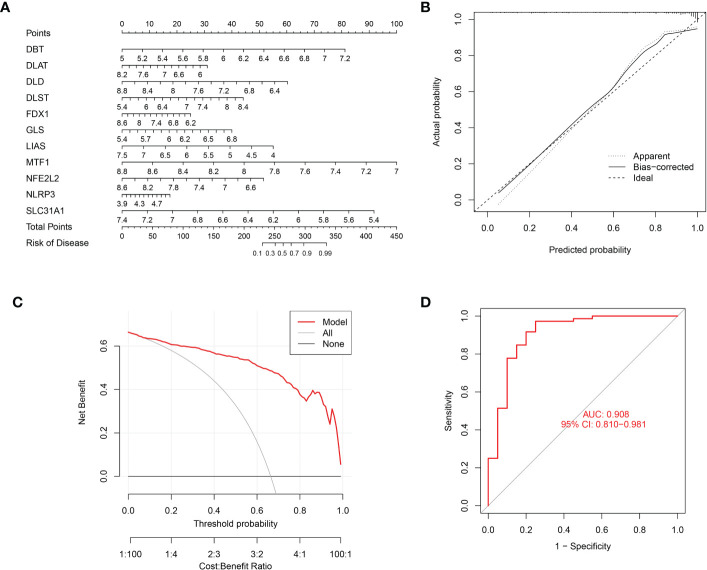
Nomogram predicting the risk in periodontitis. **(A)** Nomogram for patients with periodontitis **(B)** The calibration curve of nomogram; **(C)** The Decision curve analysis of nomogram; **(D)** ROC curve, AUC of 0.908.

A nomogram incorporating the above 11 cuproptosis genes was built (**Figure 6A**). This nomogram’s calibration curve for the disease’s deterioration risk showed that it fit the model well (**Figure 6B**). The DCA analysis was used to assess the nomogram’s clinical value (**Figure 6C**). The DCA curve showed that utilizing this nomogram to forecast patients who could deteriorate is more advantageous if the threshold probability of a patient was between 60 and 99%. ROC curve was also calculated using the training cohort GSE16134 was 0.908 (**Figure 6D**). These results showed the credibility of the 11 cuproptosis diagnostic gene signature moldel in periodontitis.

### Differential expression of the signature genes and detection the expression of the CRGs

**Figure 7:**
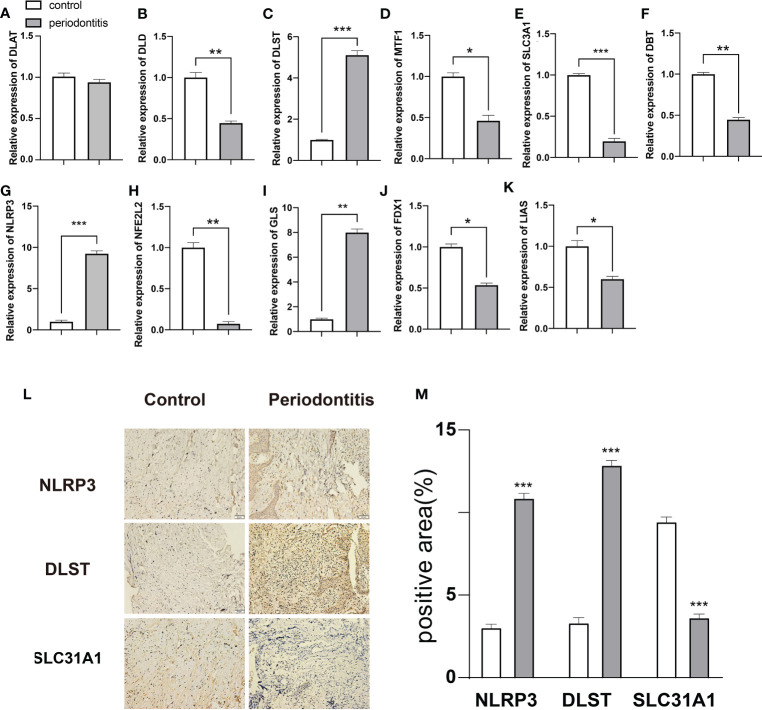
Expression analysis of 11 DE-CRGs in periodontitis and controls. **(A–K)** 11 DE-CRGs mRNA differences between the periodontitis and control groups. **(L)** Immunohistochemical staining **(M)** Histograms of quantitative immunohistochemical staining results, *p < 0.05; **p < 0.01; ***p < 0.001.

In order to further confirm the findings, we used qPCR to identify the mRNA relative expression of the 11 CRGs in 10 pairs of samples from normal and periodontitis. The results revealed that the expression of DLD, SLC31A1, DBT, DLST, NLRP3, NFE2L2, GLS, FDX1, LIAS, and MTF1 varied between normal and periodontitis. However, the expression of DLAT was not expressed differently (**Figures 7A–K**).

Then we used IHC staining to test the three most significant different genes from qPCR analysis(NLRP3, DLST and SLC3A1), evidence from IHC results showed that NLRP3 and DLST were significantly increased in the 10 periodontitis tissues than control samples. However, the expression of SLC3A1 was significantly decreased in periodontitis group (**Figures 7L, M**).

## Discussion

Periodontitis is a chronic inflammatory disease that affects the tissues surrounding and supporting the teeth, including the gums, periodontal ligament, and alveolar bone. Cuproptosis, a form of mitochondrial cell death that was originally identified in 2022, is the newest member of the controlled cell death family. It has recently been discovered to play a role in a range of disorders including neurodegeneration, cancer, and inflammation. Copper ionophores and copper chelators have already been employed in anticancer therapy (8, 24–27). Although no study has showed that periodontitis is associated with cuproptosis, there are reports dispalyed that increased risk of periodontitis was associated with excessive copper(Cu) consumption, such as the Cupral-electrophoresis approach can effectively cure destructive periodontitis in teeth with troublesome canals for up to 18 months, preserving the teeth in the process (28). According another study, severe periodontitis patients had much higher copper contents in their saliva than periodontally healthy controls (29). Additionally, it has been demonstrated that the Cu-bearing alloy improves osteoporotic macrophage responses for alveolar bone regeneration in osteoporosis patients (30).

We found that 11 CRGs expression were decresed in periodontitis, including SLC31A1, LIPT1, NFE2L2, FDX1, LIAS, ATP7A, DLD, MTF1, DLAT, DBT and PDHB, while 4 CRGs expression were increased in periodontitis, including NLRP3, GLS, CDKN2A and DLST. We also used qPCR ana IHC staining to confirm the the 11 CuGs relative mRNA expression in 10 pairs of control and periodontitis tissues. However, how these genes regulate the cuproptosis in periodontitis need to be confirmed by further *in vivo* and *in vitro* experiments.

Then the optimum parameter and SVM-RFE algorithm were used to identify the potential marker to diagnose periodontitis. 11 genes (NFE2L2, NLRP3, SLC31A1, FDX1, LIAS, DLD, DLAT, MTF1, GLS, DBT, DLST) were acquired by the two algorithms and may served as genes to diagnose periodontitis. A subsequent ROC analysis revealed that all hub genes were crucial in the development of periodontitis, suggesting a potential role for diagnosis in clinical therapy. The AUC values of the 11 CRGs were 0.905 showed that the logistic regression model built based on these 11 marker genes are well distinguished periodontitis from controls.

Compared with normal samples, plasma cells, navie CD4^+^ T cells, memory activated CD4^+^ T cells, gamma delta T cells, activated NK cells, Eosinophils Mast cells activated, M0 Macrophages and Neutrophils showed higher infiltration levels, while activated memory B cells, CD8^+^ T cells, M1 Macrophages, follicular helper T cells, resting Dendritic cells, resting mast cell displayed a lower infiltration level. In addition, it was discovered that the ratio of immune cells and the expression of these 11 genes indicated that they were closely related to dormant Dendritic cells, resting Mast cells and plasma cells, which demonstrated that immune expression was aggravated in periodontitis tissues.

In this study, we constructed three distinct cuproptosis clusters subtypes in periodontitis based on unsupervised consensus cluster analysis of the 11 marker regulators. Surprisingly, GSVA analysis found that cluster 2 was more related to various immune-related signaling pathways and cells. As we found that the cuproptosis score is an independent predictor of periodontitis. The calibration curves revealed that there was very little difference between the real and predicted periodontitis risks, indicating that the column line graph model of periodontitis is quite accurate. The nomogram model also shown good reliability and validity after evaluation. Also ROC curves of the 11 hub genes constructed by using the validation datasets (GSE16134) also corroborated the findings showed that these 11 genes played an important role in the pathogenesis of periodontitis. We also verify the expression of 11 hub genes in periodontitis patients and control samples by using qRT-PCR and IHC staining, the results showed that DLST, NLRP3 and GLS were highly expressed in periodontitis samples, while the expression of DLD, SLC31A1, DBT, NFE2L2, FDX1, LIAS and MTF1 were decreased in periodontitis samples.The expression of DLAT did not show any difference in periodontitis and control samples. Among these genes NLRP3 and NFE2L2 related with periodontitis were reported. NLRP3 complex inflammasome is very important in the development of periodontitis and diabetes (31). Chronic inflammation, oxidative stress, and NFE2L2 signaling failure in the periodontium are all potential causes of alveolar bone resorption, which can progress to periodontitis (32). The other genes among 11 hub genes have not been dicovered to be related to periodontitis.

However, they were related to oxidative stress and inflammation, which contribute a lot to the development periodontitis. Such as reduction of DLST can increase reactive oxygen species production (33), GLS is encoded by two humans genes GLS1 and GLS2, GLS2 can control the availability of energy and defend against oxidative stress (34). DLD is a highly conserved multifunctional mitochondrial enzyme and can function as a diaphorase, using NADH to generate reactive oxygen species (ROS) (35). DBT can promote oxidative stress and increase inflammatory mediators in BV-2 microglia cells (36). We think that cuproptosis may also affect the development of periodontitis by exacerbating oxidative stress and inflammation.

In conclusion, these findings have important implications for the cuproptosis and periodontitis, and highlight further research is needed to better understand the mechanisms underlying this relationship between the cuproptosis and periodontitis. As a result, the findings that have been given must be viewed as preliminary and used to guide further investigation.

## Data availability statement

The datasets presented in this study can be found in online repositories. The names of the repository/repositories and accession number(s) can be found in the article/**Supplementary Material**.

## Ethics statement

The studies involving human participants were reviewed and approved by The Medical Ethics Committee of Children’s Hospital of Nanjing Medical University. The patients/participants provided their written informed consent to participate in this study.

## Author contributions

JZ, AG and JW designed studies and revised manuscripts. SL, JG and YC carried out bioinformatical analysis and experiments. SC collected the samples and performed the statistical analysis. SL and JG performed the statistical analysis and drafted manuscripts. YC revised manuscripts. All authors contributed to the article and approved the submitted version.
