# Corrigendum: Identification of hub cuproptosis related genes and immune cell infiltration characteristics in periodontitis

**DOI:** 10.3389/fimmu.2023.1281051

**Published:** 2023-10-03

**Authors:** Shuying Liu, Jiaying Ge, Yiting Chu, Shuangyu Cai, Aixiu Gong, Jun Wu, Jinghan Zhang

**Affiliations:** ^1^ Department of Stomatology, Children’s Hospital of Nanjing Medical University, Nanjing, Jiangsu, China; ^2^ Department of Anatomy, Histology and Embryology, Nanjing Medical University, Nanjing, Jiangsu, China; ^3^ Department of Obstetrics and Gynecology, Nanjing Drum Tower Hospital, Nanjing University Medical School, Nanjing, Jiangsu, China

**Keywords:** cuproptosis, hub, immune, infiltration, periodontitis

In the published article, there was an error in affiliation 3. Instead of “Neonatal Medical Center, Children’s Hospital of Nanjing Medical University, Nanjing, Jiangsu, China”, it should be “Department of Obstetrics and Gynecology, Nanjing Drum Tower Hospital, Nanjing University Medical School, Nanjing, Jiangsu, China”.

In the published article, there was an error in the author list. Author Aixiu Gong should have appeared before author Jun Wu. The corrected author list appears below.

Shuying Liu^1^†, Jiaying Ge^2^†, Yiting Chu^1^†, Shuangyu Cai^2^, Aixiu Gong^1^*, Jun Wu^2^* and Jinghan Zhang^3^*.

The authors apologize for these errors and state that they do not change the scientific conclusions of the article in any way. The original article has been updated.

