# Supplementary material for: Identification of hub cuproptosis related genes and immune cell infiltration characteristics in periodontitis
Source: Front Immunol. 2023 May 5;14:1164667. doi: 10.3389/fimmu.2023.1164667 (PMC10196202; doi:10.3389/fimmu.2023.1164667)
Supplement: Supplementary file 1 [file Table_1.docx]

Supplementary Table 1: The primer sequences for RT-qPCR

| Primer name | Sequence |
| --- | --- |
| DLAT（F） | CCGCCGCTATTACAGTCTTCC |
| DLAT（R） | CTCTGCAATTAGGTCACCTTCAT |
| DLD（F） | CTCATGGCCTACAGGGACTTT |
| DLD（R） | GCATGTTCCACCAAGTGTTTCAT |
| DLST（F） | GAACTGCCCTCTAGGGAGAC |
| DLST（R） | AACCTTCCTGCTGTTAGGGTA |
| MTF1（F） | CACAGTCCAGACAACAACATCA |
| MTF1（R） | GCACCAGTCCGTTTTTATCCAC |
| SLC3A1（F） | CAGGAGCCCGACTTCAAGG |
| SLC3A1（R） | GAGGGCAATGATGGCTATGGT |
| DBT（F） | CAGTTCGCCGTCTGGCAAT |
| DBT（R） | CCTGTGAATACCGGAGGTTTTG |
| NLRP3（F） | CGTGAGTCCCATTAAGATGGAGT |
| NLRP3（R） | CCCGACAGTGGATATAGAACAGA |
| NFE2L2（F） | TCAGCGACGGAAAGAGTATGA |
| NFE2L2（R） | CCACTGGTTTCTGACTGGATGT |
| GLS（F） | AGGGTCTGTTACCTAGCTTGG |
| GLS（R） | ACGTTCGCAATCCTGTAGATTT |
| FDX1（F） | TTCAACCTGTCACCTCATCTTTG |
| FDX1（R） | TGCCAGATCGAGCATGTCATT |
| LIAS（F） | CAGCCCAGTCAGACCGTTAAG |
| LIAS（R） | TTTCTGGCGTTTTAGGTTTCCT |
